# Poly[aqua­[μ-*N*′-(carboxymethyl)ethylene­diamine-­*N*,*N*,*N*′-triacetato]neodymium(III)]

**DOI:** 10.1107/S1600536808026445

**Published:** 2008-08-23

**Authors:** Xiao-Hui Huang, Xiao-Hong Xu, Wei-Bo Pan, Rong-Hua Zeng

**Affiliations:** a School of Chemistry and the Environment, South China Normal University, Guangzhou 510006, People’s Republic of China; b Key Laboratory of Electrochemical Technology of Energy Storage and Power Generation in Guangdong Universities, Guangzhou 510631, People’s Republic of China

## Abstract

In the title complex, [Nd(C_10_H_13_N_2_O_8_)(H_2_O)]_*n*_, each Nd^III^ ion is coordinated by six O atoms and two N atoms from one *N*′-(carboxymethyl)ethylene­diamine-­*N*,*N*,*N*′-triacetate (edta) ligand and one water mol­ecule, displaying a bicapped trigonal-prismatic geometry. The edta ligands link the neodymium metal centres, forming polymeric chains running along the *a* axis of the unit cell. These chains are further assembled *via* inter­molecular O—H⋯O hydrogen-bonding inter­actions into a three-dimensional supra­molecular network.

## Related literature

For related literature, see: Moulton & Zaworotko (2001 ▶); Zeng *et al.*, (2007 ▶).
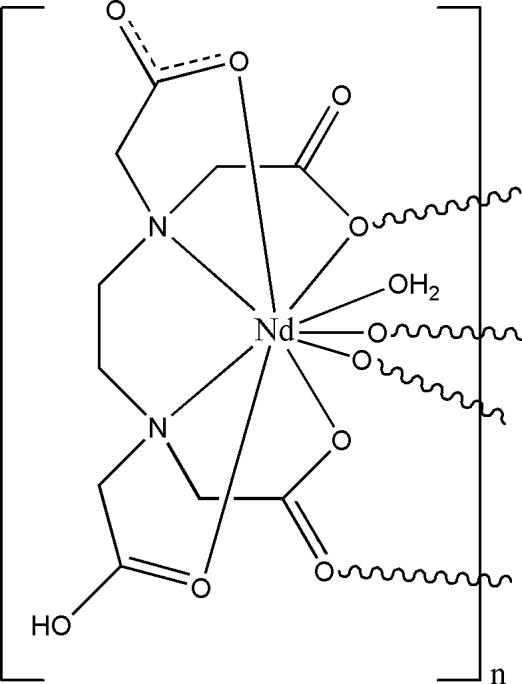


## Experimental

### 

#### Crystal data

[Nd(C_10_H_13_N_2_O_8_)(H_2_O)]
*M* *_r_* = 451.48
Orthorhombic, 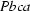

*a* = 6.6420 (3) Å
*b* = 14.7273 (6) Å
*c* = 26.0161 (10) Å
*V* = 2544.86 (18) Å^3^
*Z* = 8
Mo *K*α radiation
μ = 4.14 mm^−1^
*T* = 296 (2) K
0.22 × 0.20 × 0.19 mm

#### Data collection

Bruker APEXII area-detector diffractometer
Absorption correction: multi-scan (*SADABS*; Sheldrick, 1996 ▶) *T*_min_ = 0.42, *T*_max_ = 0.46
22432 measured reflections
3028 independent reflections
2647 reflections with *I* > 2σ(*I*)
*R*_int_ = 0.034

#### Refinement

*R*[*F* ^2^ > 2σ(*F* ^2^)] = 0.019
*wR*(*F* ^2^) = 0.041
*S* = 1.02
3028 reflections
200 parameters
3 restraints
H-atom parameters constrained
Δρ_max_ = 0.71 e Å^−3^
Δρ_min_ = −0.55 e Å^−3^

### 

Data collection: *APEX2* (Bruker, 2004 ▶); cell refinement: *APEX2*; data reduction: *APEX2*; program(s) used to solve structure: *SHELXS97* (Sheldrick, 2008 ▶); program(s) used to refine structure: *SHELXL97* (Sheldrick, 2008 ▶); molecular graphics: *SHELXTL* (Sheldrick, 2008 ▶); software used to prepare material for publication: *SHELXL97*.

## Supplementary Material

Crystal structure: contains datablocks I, global. DOI: 10.1107/S1600536808026445/bg2202sup1.cif

Structure factors: contains datablocks I. DOI: 10.1107/S1600536808026445/bg2202Isup2.hkl

Additional supplementary materials:  crystallographic information; 3D view; checkCIF report

## Figures and Tables

**Table 1:** Selected bond lengths (Å)

Nd1—O1	2.3675 (16)
Nd1—O5	2.4078 (18)
Nd1—O6^i^	2.4173 (17)
Nd1—O7^ii^	2.4892 (16)
Nd1—O7	2.4932 (16)
Nd1—O3	2.5377 (16)
Nd1—O1*W*	2.5516 (18)
Nd1—N1	2.7484 (19)
Nd1—N2	2.803 (2)

Symmetry codes: (i) 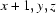; (ii) 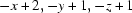.

**Table 2:** Hydrogen-bond geometry (Å, °)

*D*—H⋯*A*	*D*—H	H⋯*A*	*D*⋯*A*	*D*—H⋯*A*
O1*W*—H2*W*⋯O8^iii^	0.85	2.10	2.941 (3)	174
O1*W*—H1*W*⋯O5^ii^	0.85	1.96	2.778 (2)	162
O4—H4⋯O2^iv^	0.82	1.67	2.475 (2)	166

Symmetry codes: (ii) 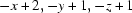; (iii) 
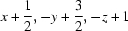
; (iv) 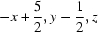.

**Fig. 1.**
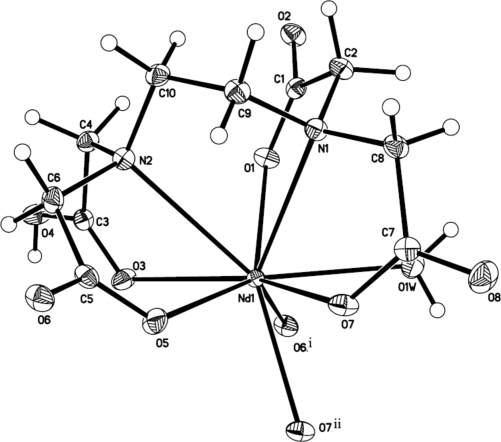
The molecular structure showing the atomic-numbering scheme. Displacement ellipsoids drawn at the 30% probability level. Symmetry codes: (i)1 + x, y, z; (ii)2 - x, 1 - y, 1 - z.

**Fig. 2.**
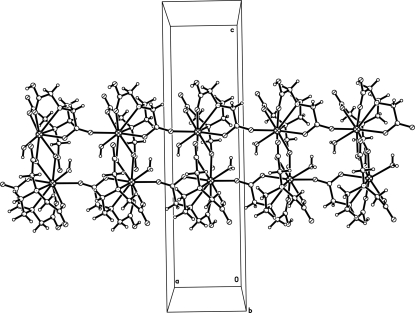
View of an infinite polymeric chain of the title complex.

## supplementary crystallographic information

### Comment

Molecular self-assembly of supramolecular architectures has received much attention during recent decades (Zeng *et al.*, 2007; Moulton & Zaworotko, 2001). The structures and properties of such systems depend on the coordination and geometric preferences of both the central metal ions and the bridging building blocks, as well as the influence of weaker non-covalent interactions, such as hydrogen bonds and π-π stacking interactions. Recently, we obtained the title coordination polymer, which was synthesized under hydrothermal conditions.

As illustrated in Fig. 1, each Nd^III^ centre is in a bicapped trigonal prismatic geometry, defined by six oxygen and two nitrogen atoms from one ethylene-diamine tetraacetate(edta) ligand  and one water molecule. The Nd^III^ ions are linked by edta ligands to form an infinite polymeric chain in the *a* axis direction(Fig.2), and the adjacent Nd···Nd separations are 4.253 (4) and 6.642 (5) Å, respectively. O—H···O hydrogen bonding (Table 1), involving carboxylate groups of edta ligands and the coordinating water molecules, assemble neighbouring chains to form a three-dimensional supramolecular network motif.

### Experimental

A mixture of Nd_2_O_3_ (0.168 g; 0.5 mmol), ethylene-diamine tetraacetic acid (0.292 g; 1 mmol), water (10 ml) in the presence of HNO_3_ (0.1 mmol) was stirred vigorously for 20 min and then sealed in a Teflon-lined stainless-steel autoclave (15 ml, capacity). The autoclave was heated to and maintained at 433 K for 3 days, and then cooled to room temperature at 5 K h^-1^ to obtain the colorless block crystals.

### Refinement

Water H atoms were tentatively located in difference Fourier maps and were refined with distance restraints of O–H = 0.84 Å and H···H = 1.35 Å, and with *U*_iso_(H) = 1.5 *U*_eq_(O), and then were treated in riding mode. Carbon-bound H and the carboxylate H4 atoms were placed at calculated positions and were treated as riding on the parent  atoms with C—H = 0.97 Å, O4-H4: 0.82 and *U*_iso_(H) = 1.2 *U*_eq_(parent).

### Crystal data

**Table:**

[Nd(C_10_H_13_N_2_O_8_)(H_2_O)]	*F*_000_ = 1768
*M**_r_* = 451.48	*D*_x_ = 2.357 Mg m^−^^3^
Orthorhombic, *P**b**c**a*	Mo *K*α radiation λ = 0.71073 Å
Hall symbol: -P 2ac 2ab	Cell parameters from 7620 reflections
*a* = 6.6420 (3) Å	θ = 2.8–27.8º
*b* = 14.7273 (6) Å	µ = 4.14 mm^−^^1^
*c* = 26.0161 (10) Å	*T* = 296 (2) K
*V* = 2544.86 (18) Å^3^	Block, colourless
*Z* = 8	0.22 × 0.20 × 0.19 mm

### Data collection

**Table:**

Bruker APEXII area-detector diffractometer	3028 independent reflections
Radiation source: fine-focus sealed tube	2647 reflections with *I* > 2σ(*I*)
Monochromator: graphite	*R*_int_ = 0.034
*T* = 296(2) K	θ_max_ = 27.8º
φ and ω scans	θ_min_ = 1.6º
Absorption correction: multi-scan(SADABS; Sheldrick, 1996)	*h* = −7→8
*T*_min_ = 0.42, *T*_max_ = 0.46	*k* = −19→19
22432 measured reflections	*l* = −32→34

### Refinement

**Table:**

Refinement on *F*^2^	Secondary atom site location: difference Fourier map
Least-squares matrix: full	Hydrogen site location: inferred from neighbouring sites
*R*[*F*^2^ > 2σ(*F*^2^)] = 0.019	H-atom parameters constrained
*wR*(*F*^2^) = 0.041	*w* = 1/[σ^2^(*F*_o_^2^) + (0.016*P*)^2^ + 2.21*P*] where *P* = (*F*_o_^2^ + 2*F*_c_^2^)/3
*S* = 1.02	(Δ/σ)_max_ = 0.004
3028 reflections	Δρ_max_ = 0.71 e Å^−^^3^
200 parameters	Δρ_min_ = −0.54 e Å^−^^3^
3 restraints	Extinction correction: none
Primary atom site location: structure-invariant direct methods	

### Special details

**Table:**

Geometry. All e.s.d.'s (except the e.s.d. in the dihedral angle between two l.s. planes) are estimated using the full covariance matrix. The cell e.s.d.'s are taken into account individually in the estimation of e.s.d.'s in distances, angles and torsion angles; correlations between e.s.d.'s in cell parameters are only used when they are defined by crystal symmetry. An approximate (isotropic) treatment of cell e.s.d.'s is used for estimating e.s.d.'s involving l.s. planes.
Refinement. Refinement of *F*^2^ against ALL reflections. The weighted *R*-factor *wR* and goodness of fit *S* are based on *F*^2^, conventional *R*-factors *R* are based on *F*, with *F* set to zero for negative *F*^2^. The threshold expression of *F*^2^ > σ(*F*^2^) is used only for calculating *R*-factors(gt) *etc*. and is not relevant to the choice of reflections for refinement. *R*-factors based on *F*^2^ are statistically about twice as large as those based on *F*, and *R*- factors based on ALL data will be even larger.

### Fractional atomic coordinates and isotropic or equivalent isotropic displacement parameters (Å^2^)

**Table:**

	*x*	*y*	*z*	*U*_iso_*/*U*_eq_	
Nd1	1.075731 (17)	0.492979 (7)	0.420675 (4)	0.01307 (4)	
C1	1.2133 (4)	0.64721 (15)	0.33465 (8)	0.0191 (5)	
C2	1.0901 (4)	0.71297 (15)	0.36663 (9)	0.0223 (5)	
H2A	1.1805	0.7498	0.3872	0.027*	
H2B	1.0168	0.7533	0.3438	0.027*	
C3	1.0295 (4)	0.38164 (15)	0.30802 (9)	0.0202 (5)	
C4	0.9158 (4)	0.46577 (16)	0.29163 (9)	0.0206 (5)	
H4A	1.0085	0.5096	0.2770	0.025*	
H4B	0.8172	0.4501	0.2656	0.025*	
C5	0.5878 (4)	0.44098 (15)	0.40469 (9)	0.0195 (5)	
C6	0.6307 (4)	0.45104 (17)	0.34798 (9)	0.0226 (5)	
H6A	0.6460	0.3911	0.3331	0.027*	
H6B	0.5158	0.4797	0.3317	0.027*	
C7	0.9063 (4)	0.66244 (15)	0.49694 (9)	0.0193 (5)	
C8	0.9167 (4)	0.71960 (15)	0.44896 (9)	0.0223 (5)	
H8A	0.7933	0.7544	0.4461	0.027*	
H8B	1.0271	0.7623	0.4524	0.027*	
C9	0.7466 (4)	0.65605 (15)	0.37536 (9)	0.0219 (5)	
H9A	0.6927	0.7157	0.3676	0.026*	
H9B	0.6542	0.6264	0.3989	0.026*	
C10	0.7579 (4)	0.60141 (15)	0.32623 (9)	0.0213 (5)	
H10A	0.6283	0.6031	0.3091	0.026*	
H10B	0.8565	0.6286	0.3034	0.026*	
N1	0.9454 (3)	0.66667 (13)	0.40105 (7)	0.0189 (4)	
N2	0.8145 (3)	0.50536 (12)	0.33635 (7)	0.0186 (4)	
O1	1.2294 (3)	0.56652 (10)	0.34990 (6)	0.0231 (4)	
O2	1.2979 (3)	0.67575 (11)	0.29451 (6)	0.0268 (4)	
O3	1.0728 (3)	0.36869 (11)	0.35312 (6)	0.0257 (4)	
O4	1.0749 (3)	0.32789 (12)	0.27038 (6)	0.0323 (5)	
H4	1.1285	0.2819	0.2817	0.048*	
O5	0.7375 (3)	0.43953 (12)	0.43463 (6)	0.0263 (4)	
O6	0.4103 (3)	0.42845 (11)	0.41880 (6)	0.0240 (4)	
O7	0.9077 (3)	0.57641 (11)	0.49218 (6)	0.0236 (4)	
O8	0.9002 (3)	0.70106 (13)	0.53946 (6)	0.0317 (4)	
O1W	1.3251 (3)	0.60217 (12)	0.46245 (7)	0.0298 (4)	
H1W	1.3343	0.5883	0.4939	0.045*	
H2W	1.3550	0.6580	0.4605	0.045*	

### Atomic displacement parameters (Å^2^)

**Table:**

	*U*^11^	*U*^22^	*U*^33^	*U*^12^	*U*^13^	*U*^23^
Nd1	0.01078 (7)	0.01511 (7)	0.01332 (7)	−0.00035 (4)	0.00070 (4)	0.00100 (4)
C1	0.0148 (12)	0.0235 (11)	0.0189 (11)	−0.0010 (9)	−0.0003 (9)	0.0003 (9)
C2	0.0241 (14)	0.0185 (11)	0.0243 (12)	−0.0013 (10)	0.0044 (10)	0.0024 (9)
C3	0.0200 (13)	0.0200 (11)	0.0208 (12)	−0.0005 (9)	0.0011 (9)	−0.0013 (9)
C4	0.0230 (13)	0.0217 (11)	0.0171 (11)	0.0025 (10)	0.0002 (9)	−0.0009 (9)
C5	0.0170 (13)	0.0151 (10)	0.0265 (12)	0.0008 (9)	0.0015 (9)	0.0009 (9)
C6	0.0169 (13)	0.0260 (12)	0.0249 (13)	−0.0037 (10)	−0.0012 (10)	−0.0012 (10)
C7	0.0134 (13)	0.0227 (11)	0.0217 (12)	0.0018 (9)	0.0020 (9)	0.0018 (9)
C8	0.0290 (15)	0.0161 (11)	0.0216 (12)	0.0032 (10)	0.0035 (10)	−0.0016 (9)
C9	0.0184 (13)	0.0201 (11)	0.0271 (12)	0.0046 (9)	0.0002 (10)	0.0002 (9)
C10	0.0188 (13)	0.0216 (11)	0.0236 (12)	0.0046 (9)	−0.0028 (10)	0.0008 (9)
N1	0.0216 (12)	0.0176 (9)	0.0176 (10)	0.0010 (8)	0.0035 (8)	−0.0011 (7)
N2	0.0163 (11)	0.0199 (10)	0.0197 (10)	0.0015 (8)	0.0014 (7)	−0.0006 (8)
O1	0.0221 (10)	0.0198 (8)	0.0274 (9)	0.0024 (7)	0.0054 (7)	0.0043 (7)
O2	0.0329 (11)	0.0240 (8)	0.0236 (9)	−0.0020 (8)	0.0092 (7)	0.0018 (7)
O3	0.0324 (11)	0.0245 (9)	0.0202 (9)	0.0078 (8)	−0.0044 (7)	−0.0004 (7)
O4	0.0511 (14)	0.0255 (9)	0.0204 (9)	0.0149 (9)	0.0014 (8)	−0.0010 (7)
O5	0.0168 (10)	0.0368 (10)	0.0252 (9)	−0.0068 (8)	−0.0041 (7)	0.0059 (7)
O6	0.0149 (9)	0.0252 (9)	0.0319 (10)	−0.0015 (7)	0.0027 (7)	0.0057 (7)
O7	0.0273 (10)	0.0204 (8)	0.0231 (9)	0.0058 (7)	0.0058 (7)	0.0053 (6)
O8	0.0379 (12)	0.0382 (10)	0.0191 (9)	−0.0007 (9)	0.0046 (8)	−0.0050 (8)
O1W	0.0329 (12)	0.0320 (10)	0.0247 (10)	−0.0054 (8)	−0.0035 (8)	−0.0016 (7)

### Geometric parameters (Å, °)

**Table:**

Nd1—O1	2.3675 (16)	C5—O5	1.263 (3)
Nd1—O5	2.4078 (18)	C5—C6	1.510 (3)
Nd1—O6^i^	2.4173 (17)	C6—N2	1.490 (3)
Nd1—O7^ii^	2.4892 (16)	C6—H6A	0.9700
Nd1—O7	2.4932 (16)	C6—H6B	0.9700
Nd1—O3	2.5377 (16)	C7—O8	1.245 (3)
Nd1—O1W	2.5516 (18)	C7—O7	1.273 (3)
Nd1—N1	2.7484 (19)	C7—C8	1.507 (3)
Nd1—N2	2.803 (2)	C8—N1	1.482 (3)
C1—O1	1.257 (3)	C8—H8A	0.9700
C1—O2	1.258 (3)	C8—H8B	0.9700
C1—C2	1.517 (3)	C9—N1	1.488 (3)
C2—N1	1.480 (3)	C9—C10	1.512 (3)
C2—H2A	0.9700	C9—H9A	0.9700
C2—H2B	0.9700	C9—H9B	0.9700
C3—O3	1.223 (3)	C10—N2	1.487 (3)
C3—O4	1.295 (3)	C10—H10A	0.9700
C3—C4	1.512 (3)	C10—H10B	0.9700
C4—N2	1.465 (3)	O4—H4	0.8200
C4—H4A	0.9700	O1W—H1W	0.85
C4—H4B	0.9700	O1W—H2W	0.85
C5—O6	1.249 (3)		
			
O1—Nd1—O5	131.99 (6)	O6—C5—O5	124.0 (2)
O1—Nd1—O6^i^	76.58 (6)	O6—C5—C6	118.7 (2)
O5—Nd1—O6^i^	137.07 (6)	O5—C5—C6	117.1 (2)
O1—Nd1—O7^ii^	151.30 (6)	N2—C6—C5	113.9 (2)
O5—Nd1—O7^ii^	76.68 (6)	N2—C6—H6A	108.8
O6^i^—Nd1—O7^ii^	79.42 (5)	C5—C6—H6A	108.8
O1—Nd1—O7	123.22 (5)	N2—C6—H6B	108.8
O5—Nd1—O7	68.35 (6)	C5—C6—H6B	108.8
O6^i^—Nd1—O7	128.34 (6)	H6A—C6—H6B	107.7
O7^ii^—Nd1—O7	62.77 (6)	O8—C7—O7	122.8 (2)
O1—Nd1—O3	78.15 (6)	O8—C7—C8	118.8 (2)
O5—Nd1—O3	82.04 (6)	O7—C7—C8	118.4 (2)
O6^i^—Nd1—O3	73.11 (6)	N1—C8—C7	114.11 (18)
O7^ii^—Nd1—O3	109.57 (5)	N1—C8—H8A	108.7
O7—Nd1—O3	150.33 (6)	C7—C8—H8A	108.7
O1—Nd1—O1W	76.31 (6)	N1—C8—H8B	108.7
O5—Nd1—O1W	138.37 (6)	C7—C8—H8B	108.7
O6^i^—Nd1—O1W	70.10 (6)	H8A—C8—H8B	107.6
O7^ii^—Nd1—O1W	80.92 (6)	N1—C9—C10	113.05 (19)
O7—Nd1—O1W	70.25 (6)	N1—C9—H9A	109.0
O3—Nd1—O1W	138.98 (6)	C10—C9—H9A	109.0
O1—Nd1—N1	64.25 (6)	N1—C9—H9B	109.0
O5—Nd1—N1	92.20 (6)	C10—C9—H9B	109.0
O6^i^—Nd1—N1	130.67 (6)	H9A—C9—H9B	107.8
O7^ii^—Nd1—N1	124.42 (5)	N2—C10—C9	111.66 (18)
O7—Nd1—N1	62.54 (5)	N2—C10—H10A	109.3
O3—Nd1—N1	122.70 (5)	C9—C10—H10A	109.3
O1W—Nd1—N1	72.37 (6)	N2—C10—H10B	109.3
O1—Nd1—N2	68.18 (6)	C9—C10—H10B	109.3
O5—Nd1—N2	64.01 (6)	H10A—C10—H10B	107.9
O6^i^—Nd1—N2	125.37 (6)	C2—N1—C8	110.47 (18)
O7^ii^—Nd1—N2	140.07 (6)	C2—N1—C9	110.66 (19)
O7—Nd1—N2	105.96 (6)	C8—N1—C9	108.61 (18)
O3—Nd1—N2	60.01 (5)	C2—N1—Nd1	109.67 (13)
O1W—Nd1—N2	133.97 (5)	C8—N1—Nd1	111.94 (13)
N1—Nd1—N2	66.36 (5)	C9—N1—Nd1	105.38 (13)
O1—C1—O2	122.7 (2)	C4—N2—C10	110.73 (18)
O1—C1—C2	118.4 (2)	C4—N2—C6	108.90 (18)
O2—C1—C2	118.9 (2)	C10—N2—C6	109.86 (19)
N1—C2—C1	112.84 (18)	C4—N2—Nd1	108.14 (14)
N1—C2—H2A	109.0	C10—N2—Nd1	110.90 (13)
C1—C2—H2A	109.0	C6—N2—Nd1	108.24 (13)
N1—C2—H2B	109.0	C1—O1—Nd1	129.88 (15)
C1—C2—H2B	109.0	C3—O3—Nd1	123.62 (15)
H2A—C2—H2B	107.8	C3—O4—H4	109.5
O3—C3—O4	125.1 (2)	C5—O5—Nd1	129.48 (15)
O3—C3—C4	121.0 (2)	C5—O6—Nd1^iii^	144.68 (15)
O4—C3—C4	113.8 (2)	C7—O7—Nd1^ii^	108.69 (14)
N2—C4—C3	109.35 (18)	C7—O7—Nd1	124.50 (14)
N2—C4—H4A	109.8	Nd1^ii^—O7—Nd1	117.23 (6)
C3—C4—H4A	109.8	Nd1—O1W—H1W	107.9
N2—C4—H4B	109.8	Nd1—O1W—H2W	137.6
C3—C4—H4B	109.8	H1W—O1W—H2W	105.9
H4A—C4—H4B	108.3		

Symmetry codes: (i) *x*+1, *y*, *z*; (ii) −*x*+2, −*y*+1, −*z*+1; (iii) *x*−1, *y*, *z*.

### Hydrogen-bond geometry (Å, °)

**Table:**

*D*—H···*A*	*D*—H	H···*A*	*D*···*A*	*D*—H···*A*
O1W—H2W···O8^iv^	0.85	2.10	2.941 (3)	174
O1W—H1W···O5^ii^	0.85	1.96	2.778 (2)	162
O4—H4···O2^v^	0.82	1.67	2.475 (2)	166

Symmetry codes: (iv) *x*+1/2, −*y*+3/2, −*z*+1; (ii) −*x*+2, −*y*+1, −*z*+1; (v) −*x*+5/2, *y*−1/2, *z*.
